# Emerging Metabolic Therapies in Pulmonary Arterial Hypertension

**DOI:** 10.3390/jcm6040043

**Published:** 2017-04-04

**Authors:** Lloyd D. Harvey, Stephen Y. Chan

**Affiliations:** 1 Medical Scientist Training Program, University of Pittsburgh School of Medicine and University of Pittsburgh Medical Center, Pittsburgh, PA 15213, USA; lloydharvey@pitt.edu; 2 Division of Cardiology, Center for Pulmonary Vascular Biology and Medicine, Pittsburgh Heart, Lung, Blood, and Vascular Medicine Institute, University of Pittsburgh School of Medicine and University of Pittsburgh Medical Center, Pittsburgh, PA 15213, USA

**Keywords:** pulmonary arterial hypertension, metabolism, metabolic reprogramming, hypoxia-inducible factor, therapeutics

## Abstract

Pulmonary hypertension (PH) is an enigmatic vascular disorder characterized by pulmonary vascular remodeling and increased pulmonary vascular resistance, ultimately resulting in pressure overload, dysfunction, and failure of the right ventricle. Current medications for PH do not reverse or prevent disease progression, and current diagnostic strategies are suboptimal for detecting early-stage disease. Thus, there is a substantial need to develop new diagnostics and therapies that target the molecular origins of PH. Emerging investigations have defined metabolic aberrations as fundamental and early components of disease manifestation in both pulmonary vasculature and the right ventricle. As such, the elucidation of metabolic dysregulation in pulmonary hypertension allows for greater therapeutic insight into preventing, halting, or even reversing disease progression. This review will aim to discuss (1) the reprogramming and dysregulation of metabolic pathways in pulmonary hypertension; (2) the emerging therapeutic interventions targeting these metabolic pathways; and (3) further innovation needed to overcome barriers in the treatment of this devastating disease.

## 1. Introduction

Pulmonary hypertension (PH) is an enigmatic vascular disorder characterized by complex pulmonary vascular remodeling and subsequent increased pulmonary vascular resistance. The World Health Organization (WHO) has classified PH into five major groups based on clinical associations and histologic appearance [1,2,3]. Group I comprises a severe form of this disease that has idiopathic, heritable, and comorbid etiologies (e.g., connective tissue disorders, HIV infection, schistosomiasis, etc.), termed pulmonary arterial hypertension (PAH) [1]. PAH results from the obliteration of pulmonary arterioles, thereby causing an increase in the pulmonary vascular resistance, subsequent right ventricular hypertrophy, and culminating as right heart failure [4]. Histologically, this panvasculopathy demonstrates intimal hyperplasia, medial hypertrophy, adventitial proliferation, and pathognomonic plexiform lesions in pulmonary arterioles [5]. Groups II, III, IV, and V represent a wide variety of conditions that can cause PH, such as left heart disease, lung diseases and/or hypoxia, thromboembolic diseases, and unclear multifactorial mechanisms, respectively [1,2,3].

PH and particularly PAH are highly morbid conditions. Unfortunately, the manifestations of symptoms including shortness of breath and right heart failure often come late in the course of the disease, portending a worsened clinical prognosis. Survival rates of PAH without treatment are grim: with 68%, 48%, and 34% alive at 1, 3, and 5 years, respectively [6]. Current clinical therapies primarily improve pulmonary vasomotor tone and provide symptomatic relief and lengthening of the time to clinical worsening. However, they do not reverse or prevent the disease process. As such, there is an obvious and critical need for novel therapies capable of affecting more than vasodilation.

Tracing the historical arc of PH research reveals a foundational basis of understanding rooted in pathological manifestations within the pulmonary arterioles [7]. Early investigations focused on hypoxia and the vasoconstrictive effects on pulmonary vasculature. Under normal conditions, hypoxic pulmonary vasoconstriction (HPV) is a physiologically protective response that optimizes ventilation-perfusion matching by diverting blood flow away from alveoli with poor ventilation [8]. HPV is mediated through pulmonary arterial smooth muscle cell (PASMC) mitochondria that act as oxygen sensors capable of altering reactive oxygen species (ROS) dynamics under acute hypoxic conditions [9]. Chronic hypoxia sustains vasoconstriction and induces activation of hypoxia-inducible factor (HIF), resulting in pulmonary vascular remodeling and the development of hypoxia-induced PH [10]. Induction of HIF can also occur under normoxic conditions when aberrations of this oxygen-sensing mechanism result in its inappropriate activation and the subsequent pathogenesis of PAH. Under conditions of chronic hypoxia, HIF regulates adaptive cellular responses, namely the metabolic reprogramming of mitochondria to meet cellular bioenergetic demands [11]. Thus, as a primary adaptive response to hypoxia, it now makes sense to have expected mitochondrial metabolic reprogramming as involved in the pathogenesis of hypoxia-induced PH and PAH [12,13].

The early idea of PH being mediated through hypoxia-mediated vasoconstriction served as an impetus for therapeutic intervention with the development and use of selective vasodilatory agents. Accordingly, the mainstay of current PH pharmacotherapy relies on endothelin-1 receptor antagonists, enhancers of nitric oxide (NO) signaling, and prostacyclin analogues [14]. The paradigm has shifted, however, over time toward the notion that the origin of PH is founded instead in myriad molecular aberrations that manifest as a remodeling of the pulmonary vasculature characterized by pathological hyperproliferation and a resistance to apoptosis [4]. As such, a metabolic theory of PAH has been put forth to unify several molecular signatures of the disease around an etiologic origin within mitochondrial dysregulation and metabolic reprogramming [15].

## 2. A Metabolic Theory of PAH

The basis of this metabolic theory is founded in several molecular and cellular processes observed in both PH and cancer that rely upon the mitochondrion as the epicenter of metabolic control and regulation. Mammalian cells under hypoxic conditions demonstrate a metabolic shift away from oxidative phosphorylation toward glycolysis and lactic acid fermentation to sustain ATP production and facilitate acute survival—known as the Pasteur effect. This metabolic shift toward glycolysis—first described by Otto Warburg in cancer cells under normal oxygen tension—serves to sustain uncontrolled, neoplastic growth [16]. Similarly, the hyperproliferative, antiapoptotic, and glycolytic phenotype of the vasculature in PH has drawn comparisons to the cellular phenotype observed in cancer, collectively contributing to proliferation and resistance to apoptosis [4,17].

As our understanding of metabolic dysregulation in PH has increased, the metabolic theory of PH has expanded and evolved beyond its original premise constrained by the Warburg effect. Recent expansions of the metabolic theory have included dysregulation of a multitude of pathways, which is further complicated by evidence of genetic polymorphisms altering these responses. In addition, the anatomic epicenter of metabolic dysregulation has widened. Early investigations and theories assumed metabolic dysregulation would logically manifest at the site of disease pathology—the pulmonary vasculature. However, increasing evidence reveals aberration of metabolism within the right ventricle (RV) and perhaps in skeletal muscle, possibly suggestive of paracrine and even systemic effects in disease pathogenesis [18,19]. The significance of metabolic dysfunction beyond the pulmonary vasculature has implications in reframing our understanding of metabolism in PH. For example, cells of the pulmonary vascular microenvironment and cardiomyocytes have inherently different metabolic demands and mechanisms of energy production, namely the high reliance of cardiomyocytes on fatty acid oxidation. These metabolic principles will be important in creating a more unified and comprehensive metabolic theory of PH.

Beyond a hyperproliferative and antiapoptotic phenotype, our expanding view of metabolic dysregulation has led to the discovery of other phenotypes influenced by metabolic reprogramming in PH, such as angiogenesis, vessel stiffening and fibrosis, inflammation, cell migration, and cell identity. Beyond endothelial cells, smooth muscle cells, and cardiomyocytes—where much of the initial observations of metabolic dysfunction in PH have centered—the role of metabolic dysregulation is expanding into other cell types within the vasculature, such as infiltrating inflammatory cells of the immune system and resident tissue stem cells [20]. The temporal relationship of metabolic dysregulation and its reprogramming is another critical question to be answered. Whether metabolic aberrations occur early or late in disease pathogenesis are important in understanding the overarching breadth of metabolic dysfunction in PH.

**Figure 1:**
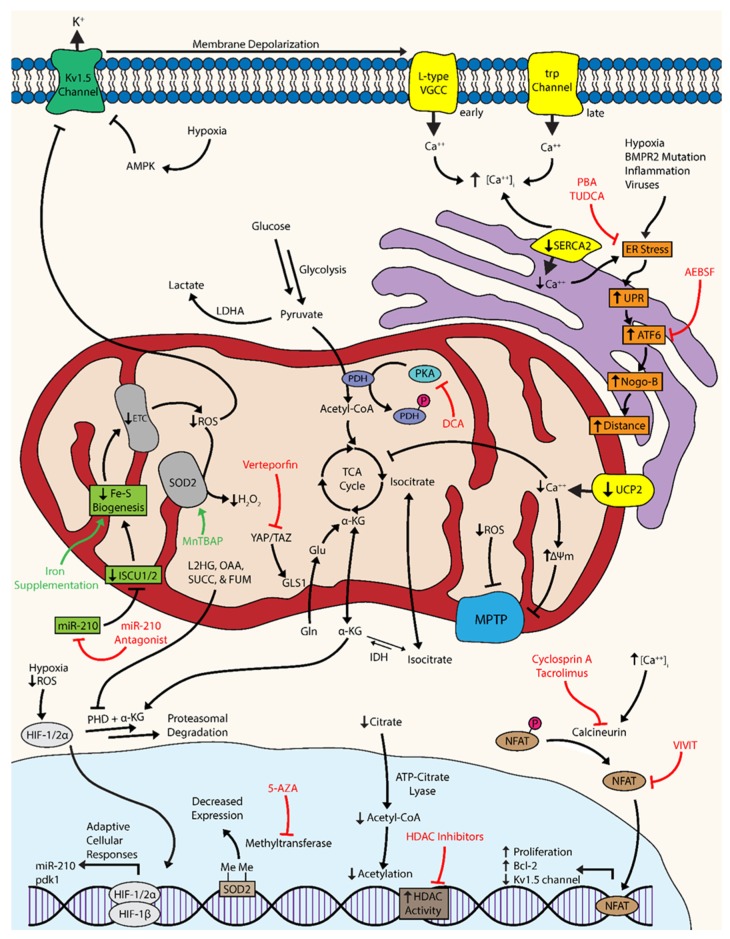
A Metabolic Theory of Pulmonary Hypertension. The activation of hypoxia-inducible factor-1/2α (HIF-1/2α) results in its nuclear translocation and binding with HIF-1β. The heterodimeric transcription factor binds to a hypoxic response element, where genes involved in adaptive cellular responses are upregulated (i.e., *pdk1* and micro-RNA 210 [miR-210]). Upregulation of pyruvate dehydrogenase kinase (PDK) results in the phosphorylation and inhibition of pyruvate dehydrogenase (PDH), limiting the flow of acetyl-CoA into the tricarboxylic acid (TCA) cycle for glucose oxidation. Instead, pyruvate is shunted via lactose dehydrogenase A (LDHA) into lactate for anaerobic respiration. HIF-mediated upregulation of miR-210 negatively regulations expression of iron-sulfur cluster assembly proteins 1 and 2 (ISCU1/2), causing a decrease in iron-sulfur biogenesis. The decrease in iron-sulfur cluster biogenesis attenuates mitochondrial respiration in the electron transport chain (ETC) and reactive oxygen species (ROS) generation. A decrease in ROS coupled with downregulation of SOD2 via methylation by methyltransferase reduces the concentration of hydrogen peroxide (H_2_O_2_), which normally inhibits HIF-1/2α activation. Decreases in ROS cause the activation of HIF-1/2α and the inhibition of Kv1.5 channels, resulting in depolarization and the activation of early current L-type voltage-gated calcium channels (VGCC) and late current transient receptor potential (trp) channels. The inhibition of Kv1.5 channels is further mediated through hypoxia-induced activation of AMP-activated protein kinase (AMPK). Increases in intracellular calcium ([Ca^++^]) inappropriately activate calcineurin, which dephosphorylates nuclear factor of activated T cells (NFAT). NFAT translocates to the nucleus where it increases proliferation, expression of bcl-2, and inhibits Kv1.5 channel expression—contributing to a self-propagating cycle. Calcium dynamics are further dysregulated with reduced activity of uncoupling protein 2 (UCP2), contributing to the dysfunction of mitochondrial enzymes and hyperpolarization of the mitochondrial membrane (ΔΨm). An increase in ΔΨm causes inhibition of the mitochondrial permeability transition pore (MPTP) and facilitates a phenotype resistant to apoptosis. A failure of the sarco-/endoplasmic reticulum calcium-ATPase 2 (SERCA2) further contributes to increased cytosolic calcium concentration. Disruption of endoplasmic reticulum (ER) calcium dynamics causes ER stress and, if prolonged, the activation of the unfolded protein response (UPR). Activating transcription factor 6 (ATF6)—a prong of the UPR—mediates the upregulation of Nogo-B, which widens the distance between the ER and mitochondrion and prevents mitochondrion-dependent apoptosis. TCA cycle intermediates such as oxaloacetate (OAA), succinate (SUCC), and fumarate (FUM) inhibit prolyl dehydrogenase (PDH)-mediated proteasomal degradation of HIF-1/2α. Decreases in the concentration of citrate reduce its nuclear conversion into acetyl-CoA by ATP-citrate lyase, thereby reducing histone acetylation. Coupled with increased histone deacetylase activity, there is a disrupted balance favoring the deacetylation of histones. Reduced levels of citrate trigger isocitrate dehydrogenase (IDH) to convert α-ketoglutarate (α-KG) into isocitrate to replenish citrate, which then deprives PHD of its cofactor necessary for hydroxylation of HIF-1/2α. Moreover, increases in the α-KG metabolite 2-hydroxyglutrate (L2HG) result in the inhibition of PHD. Activation of Yes-associated protein 1 (YAP) and transcription coactivator with a PDZ-binding motif (TAZ) upregulate glutaminase (GLS1), which converts glutamine into glutamate to ultimately replenish α-KG.

Ultimately, there are a series of coordinated levels of mitochondrial dysregulation that give rise to the cellular phenotypes of PH. The proliferation of pulmonary vascular cells, such as fibroblasts, endothelial cells (ECs), and smooth muscle cells (SMCs) are hallmarks of PH pathology [4]. The observed metabolic shift toward glycolysis has been associated with numerous molecular events that confer the ability to adapt to short bouts of cellular stress and resist apoptosis: the activation of master regulatory factors like hypoxia-inducible factor and nuclear factor of activated T cells, dysregulation of bioactive metal homeostasis, compensatory anaplerosis, a reduction in mitochondrial reactive oxygen species, the inhibition and internalization of oxygen-sensitive voltage-gated potassium channels, mitochondrial membrane hyperpolarization, dysregulation of calcium dynamics, endoplasmic reticulum stress, and the suppression of histone acetylation (Figure 1) [4,11,21,22,23]. Despite these short-term adaptations, the cell must continue to produce energy and mass at a level sufficient for metabolic demands, which typically cannot be sustained solely via enhanced glycolysis and thus requires multiple levels of molecular reprogramming.

In the context of such complexity, the therapeutic targeting of metabolism in PH confers both advantages and disadvantages. Metabolism is an exquisitely complex series of molecular events, which encompasses control of many downstream features of PH; however, metabolism is also tightly regulated in time and space, meaning that harnessing therapeutic control of metabolic pathways can be difficult and can lead to unintended side-effects. Conserved metabolic processes may allow for interventions capable of widespread targeting in various cell types and tissues, yet the efficacy of targeting a conserved pathway may be a challenge when considering systemic versus local administration of an intervention. Moreover, the intricacy inherent to metabolism poses a challenge when selecting an effector to target, which ideally has a master regulatory role in reprogramming events. Metabolic variation further serves as an obstacle in treatment with potential genetic, epigenetic, and PH-subtype differences that limit novel therapies to specific patient populations. Despite such barriers, therapeutic targeting of metabolism has great potential to treat PH early in its course, allowing for the prevention or reversal of pathologic cellular phenotypes. The targeting of novel metabolic effectors, in addition to current therapies, may provide synergistic effects in disease treatment or even reversal. A difficulty of studying efficacies of new therapies is that novel agents would be tested in patients with the current standard of care—combination therapy—further complicating clinical trials. Furthermore, new therapies could be tested in other subtypes of PH that may not lend themselves directly with the metabolic theory of PAH. In these contexts, we will discuss our understanding of the metabolic pathways involved in the development of PAH and the possibility of targeting such effectors to develop more robust diagnostics and therapeutics for this disease.

## 3. Novel Targets of Emerging Metabolic Therapies

### 3.1. Targeting Hypoxia-Inducible Factor and Downstream Metabolic Effectors

A master regulatory, oxygen sensitive transcription factor expressed in all nucleated metazoan cells—hypoxia-inducible factor (HIF)—is central to the reprogramming of metabolic activity in response to hypoxic stimulus [11]. The role of HIF in the pathogenesis of PAH is well-documented with its inappropriate induction resulting from a failure of prolyl hydroxylases (PHDs) in facilitating proteasomal degradation of the HIF-1α/HIF-2α subunit [24]. Specifically, HIF activation under normoxic or hypoxic conditions is important in the development of WHO Group I PAH and Group III hypoxia-induced PH, respectively, with possible contributions to other PH subtypes as well. The role of HIF under conditions of hypoxia and in the pathogenesis of PH has been reviewed (see [11,25,26]). In metazoan cells, hypoxic stimulus represses proteasomal degradation of the HIF-1α/HIF-2α subunit, thereby allowing for its nuclear translocation and binding with the HIF-1β subunit [26]. The resulting heterodimeric transcription factor binds to a hypoxic response element (HRE), resulting in the activation of more than a hundred genes involved in adaptive cellular responses, such as the acute metabolic shift from oxidative phosphorylation to glycolysis [26,27,28]. Studies have demonstrated the pathogenic potential of HIF-1α/HIF-2α by demonstrating protection from chronic hypoxia and resistance to the development of hypoxia-induced PH in mice heterozygous for either subunit [29,30]. Other animal models such as the fawn-hooded rat (FHR) demonstrated inappropriate HIF induction under normoxia and a predisposition to develop PAH [31,32]. Additionally, normoxic HIF activation was observed in pulmonary artery smooth muscle cells (PASMCs) from patients with PAH [31,33]. Taken together, the induction of HIF under normoxia or hypoxia lays the foundation through which subsequent molecular, cellular, and metabolic events occur in idiopathic PAH and hypoxia-induced PH, respectively.

Once activated, HIF induces transcription and expression of glycolytic genes and suppresses oxidative metabolism via increased transcription of pyruvate dehydrogenase kinase (PDK) [4]. The mitochondrial enzyme pyruvate dehydrogenase (PDH) catalyzes irreversible oxidation of pyruvate into acetyl-CoA for entry into the tricarboxylic acid (TCA) cycle and subsequent glucose oxidation (GO); however, phosphorylation of PDH by PDK results in its inhibition and a shunting of pyruvate via lactate dehydrogenase A (LDHA) into lactate for anaerobic respiration. Essentially, PDH serves as the gatekeeper that controls the rate of oxidative metabolism [34]. HIF upregulation of PDK is essential in the suppression of GO and the upregulation of glycolysis under hypoxic conditions. Under acute circumstances, this adaptive mechanism is cytoprotective, conferring short-term resistance to cellular stress; however, chronicity of environmental stressors and the dysregulation of these protective mechanisms can serve as drivers in the pathogenesis and manifestation of PH.

A mitochondrial-targeted strategy can partially correct for the resulting metabolic abnormalities observed after PDK inhibition of PDH. Dichloroacetate (DCA), an inhibitor of PDK, can attenuate the shift from oxidative phosphorylation to glycolysis by maintaining production of the acetyl-CoA necessary for the TCA cycle [35]. The use of DCA has shown promising preclinical results in the treatment of animal models of PH. One study demonstrated that DCA prevented and even reversed monocrotaline (MCT)-induced PAH in rat models via restoration of PDH activity and GO through stimulation of mitochondria-dependent apoptosis [36]. In FHRs, DCA treatment reversed normoxic HIF activation and increased voltage-gated potassium (Kv1.5) channel expression, thereby reducing PAH and improving survival [31]. In addition, this same study also inhibited HIF-1α using a selective dominant-negative construct in FHR PASMCs to show attenuation of HIF translocation and increased Kv1.5 expression [31]. Another study showed that DCA therapy in a chronic hypoxia (CH)-induced rat model enhanced activity and expression of Kv2.1 channels, improved hemodynamic parameters, and prevented and reversed CH-induced PH [21]. The culmination of such promising preclinical work was a phase I clinical trial assessing the effects of DCA treatment in patients with advanced PAH that was completed in 2013; however, the results of this study have yet to be published (NCT01083524).

### 3.2. Targeting Bioactive Metals: Iron-Sulfur, Iron, and Zinc

Bioactive metals are essential components of enzymes, functioning as cofactors necessary for metabolic reactions to occur and their dysregulation has been linked to PH. The emerging field of microRNA biology has recently shown that regulation of the metabolism is not limited to protein-coding genes, implicating a role for these small non-coding RNAs in the glycolytic shift. MicroRNA-210 (miR-210) is a known transcriptional target of activated HIF that has shown contributory regulation and facilitation of the metabolic shift toward glycolysis under hypoxic conditions [37]. Specifically, miR-210 was found to downregulate expression of the iron-sulfur cluster assembly proteins 1 and 2 (ISCU1/2) [38]. ISCU1/2 are responsible for the assembly of iron-sulfur (Fe-S) clusters—namely [4Fe-4S] and [2Fe-2S]—that are essential prosthetic groups in proper operation of the TCA cycle and mitochondrial complexes I, II, and III in the electron transport chain (ETC) [39,40]. Downregulation of ISCU1/2 by hypoxic miR-210 was found to attenuate Fe-S-dependent mitochondrial respiration in favor of glycolysis in pulmonary arterial endothelial cells (PAECs). Although this metabolic shift was protective under acute circumstances, its chronic repression drove dysfunction and reprogramming of mitochondrial metabolism, thereby promoting PH in rodent models [41]. Notably, a woman with a genetic deficiency in ISCU1/2 was found to suffer from exercise-induced pulmonary hypertension, providing important proof of the relevance of this mechanism in human disease. This principle of linking Fe-S deficiency to PH has also been supported by epidemiologic data showing the histologic manifestations of PAH in infants with genetic deficiency in NFU1, another Fe-S cluster assembly protein [42,43]. Although no small molecules have been discovered to directly regulate Fe-S cluster expression, pharmacological inhibition of upstream miR-210 with pulmonary vascular specific anti-miR delivery may serve as a promising therapeutic option as proven in rodent models of PH [41].

Iron deficiency has been observed in PAH populations, raising the notion that iron deficiency may serve as a mechanism of repressed Fe-S clusters by substrate deprivation [44,45,46,47]. Additionally, the enzymes responsible for HIF hydroxylation—like PHD—require a ferrous iron ion at their active site for electron transfer, implying that hydroxylation of HIF may be dependent on the availability of intracellular iron [48,49]. Iron-regulatory protein 1 (Irp1) binds to iron-responsive element (IRE) machinery as an apoprotein under iron-deficient conditions to regulate iron metabolism proteins. Moreover, Irp1 can also become a cytosolic aconitase in iron-replete states upon the acquisition of a [4Fe-S] cluster [50,51,52]. Thus, when hypoxia, anemia, or iron-deficient conditions are present, stabilization of HIF-2α induces erythropoietin (EPO) expression and red blood cell (RBC) development, thereby consuming iron via hemoglobin synthesis. After a certain threshold, iron deficiency halts Fe-S cluster production and Irp1 converts to the apoprotein form that binds to IREs to repress HIF-2α translation and decrease EPO production, thereby halting further iron consumption via hematopoiesis. Thus, a failure to repress HIF activation would result in its inappropriate and sustained expression as a driver in the pathogenesis of PH. Recently, it was reported that mice deficient of Irp1 (*Irp1*^−/−^) failed to repress HIF-2α translation, causing its increased expression and thus stimulated EPO synthesis to drive RBC production, iron consumption, and the development of polycythemia and PH. In vitro, PAECs from these mice showed increased expression of HIF-2α and the potent vasoconstrictor endothelin-1 [53]. Endothelin-1 binds to its receptor on SMCs to induce vasoconstriction and on cardiomyocytes and fibroblasts to induce their proliferation, which subsequently leads to PH, cardiac hypertrophy, and fibrosis [54,55]. Thus, pharmacologic activation of Irp1 binding to IREs with agents like the nitroxide Tempol may serve as therapeutic strategy in the treatment of PH [56].

Additionally, rats fed an iron-deficient diet for one month exhibited profound pulmonary vascular remodeling with upregulation of important regulators like HIF-1α and HIF-2α, decreased mitochondrial complex I activity, and upregulation of glycolytic genes like *pdk1*. Notably, the consequences of iron deficiency were reversed with iron replacement therapy via intravenous ferric carboxymaltose [57]. As such, iron replacement therapy in PAH patients with iron deficiency has been trialed with intravenous ferric carboxymaltose. The study, while it demonstrated improved exercise endurance, did not significantly alter the six-minute walk distance at 12 weeks (NCT01288651) [58]. In a recently published clinical study, iron deficiency caused exaggerated hypoxia-induced hypertension when compared to controls that could be reversed with ferric carboxymaltose administration, suggesting a role of iron deficiency in hypoxia-sensing mechanisms (NCT01847352) [59]. Other clinical trials focusing on iron homeostasis are currently underway, examining if single dose iron supplementation influences cardiopulmonary hemodynamics in PAH (NCT01447628) and if low Fe-S clusters predispose patients to the development of PH (NCT02594917). Of note, however, this biology may be context-specific, as seemingly disparate data are also emerging regarding the predisposition to PH in sickle cell patients with iron overload [60].

The dysregulation of the bioactive ion zinc has also been established in the pathogenesis of PH. Whole-genome sequencing from rats identified *Slc39a12* as a candidate gene, which encodes the solute carrier 39 zinc transporter family member 12 (ZIP12), involved in the predisposition to hypoxia-induced PH [61]. ZIP12 promotes the extracellular uptake and intracellular release of zinc in various cell types, serving as an important regulator in zinc homeostasis [62]. In hypoxia-induced PH rats, markedly increased ZIP12 mRNA levels and protein expression were observed in lung and remodeled pulmonary arterial tissues when compared to normoxic controls. Downstream of the ZIP12 transcription start site was a HRE containing HIF-1/2α binding motifs. Chromatin immunoprecipitation following hypoxic exposure in human PASMCs revealed enrichment of HIF-1/2α bound to the ZIP12 HRE, indicating a relationship with hypoxia as a driver of aberrant pathology [61]. Additionally, increased ZIP12 expression was observed in cattle with naturally occurring PH (i.e., Brisket disease) and in human populations residing above 2500 m within the remodeled pulmonary vasculature, suggesting that ZIP12 upregulation is broadly observed in response to a variety of conditions seen with chronic hypoxia. Human PASMCs exposed to hypoxia demonstrated increased ZIP12 expression, intracellular zinc, and proliferation [61]. Inhibition of ZIP12 with siRNA reversed proliferation of hypoxia-exposed human PASMCs, indicating ZIP12 involvement in cellular proliferation in response to hypoxia. Lastly, genetically ablated (*ZIP12*^−/−^) rats exposed to chronic hypoxia for two weeks had lower pulmonary arterial pressures, reduced RV hypertrophy, and less pulmonary remodeling than wild-type controls [61]. Pathologic aberrations of zinc dynamics, an ion involved in the structural components of enzymes and transcription factors, further implicated its regulation within the cell. These data cumulatively support a role for ZIP12 as a critical regulator of zinc homeostasis in the pathogenesis of PH in response to chronic hypoxic stimulus, thus suggesting its utility as a potential drug target. Taken together, targeting the dynamics of bioactive metal homeostasis, a critical regulator of metabolic events, may serve as a modulatory treatment strategy.

### 3.3. Targeting Other Tricarboxylic Acid Cycle Intermediates: Connections to Hypoxia

Aberrations of the TCA cycle and its intermediates serve to stabilize HIF and facilitate its activation. The TCA cycle-derived α-ketoglutarate (α-KG) is a required cofactor for PHD, the enzyme that under normal conditions causes the proteasomal degradation of HIF [24,63]. Other intermediates such as succinate, fumarate, and oxaloacetate inhibit the activity of PHD at high concentrations to allow translocation and activation of HIF [64]. The influence of TCA cycle intermediates also has nuclear implications with acetylation and methylation of nuclear histones both regulated by citrate and α-KG, respectively [65,66]. Citrate that leaks out of mitochondria enters the nucleus where ATP-citrate lyase creates the acetyl-CoA necessary for histone acetylation [65]. Coupled with the knowledge of a shutdown of the TCA cycle during the glycolytic shift, the notion of decreased citrate and thus decreased histone acetylation via substrate deprivation becomes an area of therapeutic interest. In addition, histone deacetylase (HDAC) activity is elevated in human PAH, indicating a potential aberration from proper epigenetic regulation via decreased histone acetylation. Accordingly, the use of the HDAC inhibitors valproic acid (VPA) and suberoylanilide hydroxamic acid (SAHA; vorinostat) were found to be effective in reversing PH in a CH-induced rat model [67]. Of note, this compensatory mechanism in the cytoplasm can synthesize citrate via the glutamine pathway, which has been established in cancer but not yet in PH [68,69].

Analyses of metabolic flux indicate that transformed cell lines may add carboxyl residues to α-KG via isocitrate dehydrogenase (IDH) to compensate for decreased levels of citrate normally derived from GO [70,71,72]. Hypoxia causes an increase in the α-KG-reduced metabolite 2-hydroxyglutarate (2HG) with both _L_ and _D_ enantiomers (L2HG and D2HG) that can inhibit PHDs [66]. A recent study showed that hypoxia in human PAECs and PASMCs specifically and dose-dependently increased L2HG in response to mitochondrial reductive stress via TCA cycle dysfunction, where it served to inhibit glycolysis and oxidative phosphorylation to regulate the cellular redox state [73]. The complexity of metabolic dysregulation clearly extends beyond the overly simplified Warburg model, requiring many more studies in understanding the elaborate metabolic network occurring within the vascular microenvironment.

As would be expected, enzymatic mutations of the TCA cycle have been associated with PAH. Mutations in the bone morphogenetic protein receptor 2 (BMPR2) are linked to the pathogenesis of PH, and expression arrays from Bmpr2 mutant mice revealed that nearly half of the significantly altered genes fell into metabolic gene ontology groups [74]. Metabolomic analysis downstream of BMPR2 mutations revealed a profound failure of anaplerosis and depletion of TCA cycle intermediates, indicating that metabolic defects in PAH are not solely due to the Warburg effect and instead are present in multiple coexisting and interdependent pathways. This study focused on the TCA cycle as the major point of metabolic integration and specifically observed that the activity of IDH is elevated in human pulmonary microvascular endothelial cells with mutant BMPR2 and in the serum of PAH patients [75]. IDH can convert α-KG into isocitrate, catalyzing against the normal flow of the TCA cycle. As such, increased reversal of IDH activity deprives PHD of its α-KG cofactor to hydroxylate HIF for proteasomal degradation and disrupts further processing of glucose for oxidation, serving as a mechanism to sustain HIF activation and translocation. In fact, increased activity of IDH has been correlated with disease severity in PAH patients [75]. The therapeutic implications of metabolomic analyses suggest that most or perhaps even all errant metabolic pathways may need to be addressed therapeutically to drive a more robust and significant improvement in clinical outcome.

### 3.4. Targeting Anaplerosis and Glutaminolysis

The metabolic shift away from oxidative phosphorylation to glycolysis alone would not be expected to serve as a favorable condition for excessive proliferation of pulmonary vascular cells. While ATP production is less efficient for glycolysis per molecule of glucose, prior studies have suggested that there is enough glucose available in the pulmonary vasculature to allow for sufficient ATP production for proliferation [22]. Independent of ATP production, however, is the concept that a decrease of carbon intermediates also results if glucose is shunted away from the TCA cycle, thus negatively affecting the carbon substrates used to produce cell mass (i.e., nucleotides and proteins). Anaplerosis, or the replenishing of TCA carbon intermediates, is therefore critically important in maintaining cell mass, particularly if glycolysis predominates. One pathway for replenishment of carbon intermediates is through the deamidation of glutamine via glutaminase (GLS1). GLS1 activity in glutaminolysis provides energy for rapidly proliferating cells, and it has been established as necessary for the formation of aspartate for malignant cell proliferation [76,77,78]. Moreover, the dysregulation of cardiac glutaminolysis has been documented in maladaptive RV hypertrophy in the MCT-induced PAH model [79]. A recent study demonstrated that two transcriptional coactivators activated by mechanotransduction—Yes-associated protein 1 (YAP) and transcriptional coactivator with a PDZ-binding motif (TAZ)—are necessary for GLS1 upregulation and subsequent glutaminolysis to sustain proliferation and migration within stiff extracellular matrix. Thus, in addition to hypoxia, this study defined a mechanical stimulus, vascular stiffness, as a metabolic mechanism by which cellular proliferation is induced in PH [80]. Importantly, the same study revealed a marked improvement in a MCT-induced PH model using a YAP inhibitor verteporfin, which is already a FDA-approved intravenous medication for age-related macular degeneration [81]. Moreover, the same effects were seen with two separate glutaminase inhibitors, one of which (CB-839) is currently under clinical investigation in human cancer trials [82] (NCT02071862). Taken together, these findings suggest that the repurposing of currently available therapies affecting vascular stiffness and glutaminolysis may have additive and perhaps synergistic effects on ameliorating or preventing PH.

### 3.5. Targeting Mitochondria-Dependent Reactive Oxygen Species and Oxidative Stress

The inhibition of pyruvate processing for aerobic respiration results in an attenuation of the TCA cycle and mitochondrial ROS generation within the ETC. Although well-known for their damaging and detrimental effects, ROS have been implicated as important signaling molecules [83], ultimately contributing to a state of pulmonary vasodilation or vasoconstriction [84]. The major sites of ROS generation are classically regarded at Complexes I and III; however, more recent studies have demonstrated other mitochondrial enzymes involved in ROS generation, such as complex II [85]. And, in vascular SMCs, the ETC has been put forth as an oxygen sensor that generates ROS in proportion to alveolar PaO_2_, suggestive of a regulatory role [86]. Accordingly, the downstream effects of decreased ROS generation cause HIF activation and the inhibition of Kv1.5 channels, leading to cellular depolarization, calcium influx via l-type voltage-gated calcium channels (VGCCs), and pulmonary vasoconstriction.

Directly related to the disruption of ROS dynamics is the corollary that manganese superoxide dismutase (Mn-SOD or SOD2), an enzyme found only in the mitochondria, is decreased in PH [87]. SOD2 converts the ROS superoxide produced in the ETC into the diffusible second messenger hydrogen peroxide (H_2_O_2_), which inhibits the activity of HIF and increases the expression and activation of Kv1.5 channels [88,89,90]. As such, decreased ROS generation coupled with low-yield transformation into H_2_O_2_ by reduced SOD2 further contributes to ionic dysregulation and downstream pulmonary vasoconstriction. One avenue of targeting SOD expression includes miR biology. The siRNA knockdown of miR-23a in human small vascular endothelial cells demonstrated significantly increased mRNA transcripts of *SOD*, suggesting that miR-23a antagonism might serve to sustain H_2_O_2_ production and its inhibition of HIF [91]. A study of patients with severe PAH noted decreased SOD2 expression and activity, further supporting a role in disease pathology [87]. Methylation of *SOD2* has been proposed as a potential epigenetic mechanism in the development in both FHRs and heritable PAH. Downregulation of SOD2 is reversible with the use of the DNA methyltransferase inhibitor 5-aza-2’-deoxycytidine (5-AZA), causing a restoration of mitochondrial function, inhibition of proliferation, and apoptosis in PAH PASMCs. The in vivo administration of the SOD-mimetic metalloporphyrin Mn(III)tetrakis (4-benzoic acid) porphyrin (MnTBAP) caused partial reversal of PAH in the FHR model [92]. In consolidating these findings, the ROS-SOD2-HIF-PDK nexus has emerged as a relevant pathogenic axis in PH, revealing multiple potential opportunities for therapy.

A recent study has implicated a role for the third isoform of SOD, which exists in the extracellular environment (EC-SOD or SOD3), in the pathogenesis of PH. SOD3 is the most abundant and active isoform within the vasculature [93]. Numerous studies and various models of lung injury or vascular injury have shown that loss of SOD3 and various polymorphisms contribute to disease severity, indicating a possible epigenetic regulatory mechanism of *SOD3* [94]. In human PASMCs, SOD3 mRNA expression was decreased in lung tissue from patients with idiopathic PAH at transplantation versus failed donors and SOD2 protein expression was unchanged, contrary to the results of a previous report [94]. In contrast to the previous work demonstrating 5-AZA effects in increasing SOD2 expression, treatment with 5-AZA did not increase PASMC SOD3 mRNA, implying that its expression is not under the regulatory control of methylation [92,94]. Class I HDAC inhibitors, however, demonstrated an increase in SOD3 and a reduction in proliferation in idiopathic PAH PASMCs, which reflects a functional connection between SOD3 regulation and class I HDACs [94].

Thus, despite some conflicting data among separate reports, the role of ROS generation and downstream metabolic signaling are clearly important components of PH pathogenesis. Whether pharmacologic targeting of SOD isoforms can be effective in treatment of PH remains to be elucidated, and to do so with predictable and limited side-effects would require several complexities in redox biology to be better defined.

### 3.6. Targeting Kv1.5 Channels and AMP Kinase in Mitochondria

A direct consequence of reduced mitochondrial ROS production is the inhibition of oxygen-sensitive, voltage-gated potassium (Kv) channels, namely Kv1.5 [95,96]. As such, the metabolic shift toward anaerobic respiration, which reduces ROS generation in the ETC, further mediates downstream effects on ion homeostasis that ultimately contributes to a self-sustaining cycle of aberrant biology. The Kv1.5 channel serves as an additional oxygen sensor for the cell via intracellular ROS detection, meaning that its inhibition can keep the cell in a (pseudo)hypoxia-induced metabolic state even if normal oxygen levels have returned. The downregulation of the Kv1.5 channel is observed in human PASMCs with PAH and rodent models of PAH [4,96,97]. The downregulation and inhibition of these channels results in a buildup of cytosolic potassium, causing depolarization of the cellular membrane and the activation of L-type VGCCs. Thus, depolarization of the membrane causes an inappropriate influx of calcium and the subsequent initiation of signaling cascades that promote pulmonary vasoconstriction and resistance to apoptosis [86]. Using a CH-induced rat model, a study demonstrated that in vivo gene transfer of Kv1.5 channels protected from the development of PH [98].

Kv1.5 channels are functionally associated with upstream metabolic effectors. Inhibition of mitochondrial oxidative phosphorylation with either phenformin or hypoxia was found to result in AMP-activated protein kinase (AMPK)-mediated inhibition of Kv1.5 channels in rat PASMCs. The same study showed that the use of various activators of AMPK also produced a marked reduction in Kv1.5 channel currents [99]. The anti-diabetic drug metformin, a known stimulator of AMPK, protected against PAH in both CH- and MCT-induced models, while also demonstrating anti-remodeling properties [100,101]. A recently published study revealed downregulation of AMPK in PAECs in both hypoxia-induced mice and PAH patients, and it showed that knockdown of endothelial AMPK promoted the development of PH in the hypoxia-induced model. Additionally, the study demonstrated that endothelial AMPK-knockdown promoted proliferation in PASMCs, suggestive of a complex relationship within the vascular microenvironment, and that the application of PAH patient serum increased proliferation of PASMCs and reduced proliferation of PAECs [102].

The pathogenic importance of AMPK signaling extends beyond PAH. Left ventricular diastolic dysfunction, or heart failure with preserved ejection fraction (HFpEF), is recognized as a clinical complication of metabolic syndrome [103,104]. As a cause of Group II PH, the relationship between HFpEF secondary to metabolic syndrome and the pathogenesis of PH is of increasing interest. The development of both metabolic syndrome and PH has been linked to the reduced production and bioavailability of nitric oxide (NO)—a known activator of AMPK [105,106,107,108,109,110]. Accordingly, treatment with nitrite or metformin at time of SU5416 injection—a VEGF receptor blocker and model of PH—in rats reduced pulmonary arterial pressures and reversed vascular remodeling [111]. Clinically, a phase 2 trial evaluating the effects of metformin in PAH at Nantes University Hospital was withdrawn prior to enrollment due difficulty in attracting patients and other universities (NCT01352026); however, a current clinical trial is actively recruiting patients to examine the effects of metformin in improving pulmonary vascular function in patients with PAH (NCT01884051).

Taken together, these recent studies suggest that either AMPK or its downstream effector Kv1.5 may serve as attractive drug targets. AMPK has great potential as a metabolic drug target in the context of the sheer quantity of molecules known to stimulate its activation, such as salicylate and methotrexate [112,113,114].

### 3.7. Targeting Calcium Ion Homeostasis and Mitochondrial Electrical Dynamics

Central to the proper metabolic functioning of mitochondria is the careful control and regulation of intramitochondrial calcium dynamics. Many of the reactions inherent to mitochondrial metabolism are highly dependent on finely-tuned calcium ion homeostasis, meaning that even slight deviations from calcium homeostasis can have profound influence on metabolic events. As is expected, intracellular and mitochondrial calcium dynamics are of great interest in the metabolic events underlying PH pathology, especially since downstream sequelae following the downregulation of Kv channels and thus retention of cytoplasmic potassium include increased intracellular calcium-mediated pulmonary vasoconstriction and cellular proliferation [36].

The intracellular calcium overload, initially driven by pathologic depolarization of the L-type VGCCs, is later maintained and reinforced by upregulation and activation of transient receptor potential (trp) channels [115,116]. Increased vascular cell proliferation—a hallmark of PAH histopathology—is in part due to activation of the calcium-calcineurin-dependent proliferative transcription factor nuclear factor of activated T cells (NFAT) [117]. The nuclear translocation of NFAT promotes proliferation and reduces expression of Kv1.5 channels in PAH PASMCs, promoting a positive feedback loop via increased intracellular potassium and subsequent depolarization-mediated calcium influx [117]. Furthermore, activation of NFAT contributes to the upregulation of the antiapoptotic protein bcl-2 (and mitochondrial hyperpolarization), which is also upregulated in human idiopathic PAH [117]. Increased expression of bcl-2 prevents mitochondrial depolarization by proapoptotic mediators via enhanced hydrogen efflux, thereby contributing to the observed hyperpolarized state [118].

As a tightly regulated ion, there exist many cellular transporters involved in maintaining calcium homeostasis. Mitochondrial calcium dynamics are regulated by a calcium-uniporter called uncoupling protein 2 (UCP2) that, unlike its name implies, moves calcium from the ER into mitochondria [119,120]. Abnormalities of UCP2 would presumably cause a deficiency in mitochondrial calcium influx from the ER, resulting in suppression of GO and induce a pathophysiological state characterized by proliferation. Furthermore, a description of polymorphisms in *UCP2* causing decreased gene expression suggests this may be a clinically relevant target [121]. Genetic ablation of *UCP2* in PASMCs demonstrated mitochondrial hyperpolarization, decreased activity of calcium-sensitive mitochondrial enzymes, resistance to apoptosis, and established a proliferative phenotype. The same study showed that in vivo knockout of *Ucp2* in mice models activated HIF-1α and NFAT, increased remodeling of the pulmonary vasculature, and developed PH [122,123]. In ECs, the loss of UCP2 resulted in exaggerated PTEN-induced putative kinase 1 (Pink1)-induced mitophagy, decreased mitochondrial biosynthesis, and increased apoptosis, indicating that a UCP2-Pink1 axis might serve as a therapeutic target [124]. Additionally, impaired function of another calcium uniporter—the mitochondrial calcium uniporter complex (MCUC)—through its downregulation decreases mitochondrial calcium levels that inhibits PDH and GO, causing the PAH phenotype in PASMCs. The in vivo administration of anti-miR-25 and anti-miR-138 restored MCUC expression and reversed established PAH in a MCT-induced rat model [125].

Intracellular calcium dynamics are also dysregulated at the level of the sarco-/endoplasmic reticulum calcium-ATPase (SERCA), a transporter that sequesters calcium into the sarcoplasmic reticulum after its depletion during excitation-contraction coupling [126]. In rat vascular injury models, the SERCA2 transporter is downregulated in the vascular media, causing increased intracellular calcium and subsequent NFAT-mediated vascular SMC proliferation and neointima formation [127]. SERCA2 expression is also decreased in human PAH PASMCs in comparison to controls, which indicates it as a target for disease treatment. Gene transfer of human SERCA2a using an adeno-associated virus serotype 1 (AAV1.SERCA2a) via aerosolized inhalation in both a MCT-induced rat PAH model and a pulmonary vein banding porcine model demonstrated increased expression of SERCA2 in pulmonary arteries, decreased pulmonary artery pressure, and improved RV function [128,129].

A further consequence of metabolically dysregulated ion homeostasis alters the electrical dynamics of the cell and ultimately mitochondria. The resistance to apoptosis observed in PAH results in part from a failure of proapoptotic mediators to efflux from mitochondria through the mitochondrial permeability transition pore (MPTP) [130]. The MPTP is a voltage- and redox-sensitive channel that closes under hyperpolarized mitochondrial membrane potential, thereby limiting the efflux of proapoptotic mediators and establishing a state of apoptotic resistance [131,132]. Inhibition of PDH prevents the movement of pyruvate into the mitochondria as acetyl-CoA for GO, thereby triggering glycolytic compensatory mechanisms. Increased activity of hexokinase-II (HK-II) during the glycolytic shift inhibits a portion of the MPTP—the voltage-dependent anion channel (VDAC) [130]. Inhibition of the VDAC causes retention of anions and hyperpolarization of the mitochondrial membrane, further limiting the function of the MPTP and inducing a relative state of apoptotic resistance [133]. Inhibition of Akt causes the activation glycogen synthase kinase 3β (GSK3β) and its translocation to the outer mitochondrial membrane, where it disrupts HK-II interaction with the VDAC preventing its inhibition [130]. As such, GSK3β prevents nuclear translocation of NFAT that upregulates anti-apoptotic mediators and downregulates Kv1.5 channel expression, further propagating and sustaining a reactive cycle. Accordingly, prevention of NFAT translocation, either directly or through other mechanisms such as the stabilization of calcium dynamics, may be a promising target for therapeutic intervention. Maintenance of proper calcium dynamics and/or prevention of NFAT translocation would prevent the upregulation of antiapoptotic molecules, the hyperpolarization of the mitochondrial membrane and thus MPTP closure, and the downregulation of Kv1.5 channels, thereby halting the metabolically-induced state of pro-proliferation and resistance to apoptosis.

The use of nonspecific calcineurin inhibitors that prevent the dephosphorylation and thus translocation of NFAT, such as cyclosporin A and tacrolimus, are current therapeutic candidates. A selective NFAT peptide inhibitor, VIVIT, specifically inhibits the docking of calcineurin onto NFAT instead of broad calcineurin inhibition, thereby limiting the adverse effects of nonspecific inhibitors [134]. One study demonstrated inhibition of NFAT using cyclosporin A or VIVIT in vitro increased Kv1.5 expression, reduced intracellular potassium and calcium levels, decreased mitochondrial membrane potential, and attenuated expression of bcl-2 [117]. The use of cyclosporin A in MCT-induced rat model produced a decrease in pulmonary vascular resistance and pulmonary arterial pressure, while increasing cardiac output [117].

Additionally, administration of low-dose tacrolimus restored signaling through the BMPR2, and reversed severe PAH in the MCT-induced model and in the VEGF receptor blockade/chronic hypoxia model, suggesting potential clinical benefit with low-dose therapy [135]. Loss of function mutations in BMPR2 are well-documented in sporadic and familial PAH [3]. Mutations in BMPR2 signaling have various roles in the pathogenesis of PH, exerting modulatory influence in certain aspects of metabolism. For example, in mice with a smooth muscle cell-specific overexpression of BMPR2, there is an observed downregulation of Kv1.5 channel expression resulting in PASMC depolarization and vasoconstriction, indicating Kv1.5 channel regulation as downstream of BMPR2 signaling [136]. Accordingly, the safety and efficacy of low-dose tacrolimus was trialed in a phase II study (NCT01647945) that has yet to be published; however, the authors recently reported a case of three patients with end-stage PAH who, ineligible for enrollment, instead received compassionate low-dose tacrolimus. Although the data are preliminary, the authors note a marked stabilization in cardiac function and freedom from hospitalization due to RV failure [137].

### 3.8. Targeting Mitochondrial Fission, Fusion, and Function

Mitochondrial networks within the cell represent a dynamic balance between fusion and fission. During cell division, mitochondrial fission evenly partitions mitochondria into daughter cells, whereas mitochondrial fusion is the joining of mitochondria to create an interconnected network for the distribution of mitochondrial proteins and DNA [138,139]. In hyperproliferative states, mitochondrial fission is necessary to sustain daughter cell production, which identifies this process as a possible intervention. Accordingly, the activity of dynamin-related protein-1 (DRP1)—a GTPase that regulates mitochondrial fission and fragmentation via the formation of oligomeric complexes and mechanical constriction of mitochondria—is of interest [140,141]. DRP1 is activated via phosphorylation by cyclin B1/cyclin-dependent kinase 1 (CDK1) at serine 616, causing its translocation to the cytoplasm for mitochondrial fragmentation [142]. Conversely, phosphorylation by protein kinase A at serine 637 results in its inhibition [143,144].

It has been reported that HIF-1α activation in human PAH PASMCs resulted in mitochondrial fission via cyclin B1/CDK1 phosphorylation of DRP1, in comparison to human control PASMCs that could be stimulated to undergo DRP1-mediated fission through chemical activation of HIF-1α [142]. The same study demonstrated that the inhibition of DRP1 with the small molecule Mdivi-1 reduced its activation, mitochondrial fission, and PASMC proliferation. Furthermore, in vivo administration of Mdivi-1 inhibited proliferation, while improving exercise capacity and RV function. Thus, the hyperproliferative phenotype characteristic in PAH, which is sustained through metabolic reprogramming, can also be targeted by locking mitochondria into a homogenous network, thereby causing cell-cycle arrest and preventing proliferation.

Decreased activation of the peroxisome proliferator-activated receptor-γ coactivator 1α (PGC1α), a transcription factor involved in mitochondrial biogenesis, is associated with increased mitochondrial fission [145]. In both EC and SMCs, BMPR2 regulates the peroxisome proliferator-activated receptor-γ (PPARγ)-mediated gene transcription [146,147]. Thus, a dysfunction in PGC1α may serve a key role in the biogenesis of mitochondria. Accordingly, loss of BMPR2 in human PAECs under normoxia increased PGC1α transcriptional activity, mitochondrial ATP production, glycolysis, and mitochondrial fission [148]. However, during hypoxia-reoxygenation, a reduction in BMPR2 demonstrated decreased mitochondrial biogenesis and energy metabolism that promoted EC apoptosis, which has been linked to SMC proliferation due to the loss of EC repressive growth factors [148,149,150]. The loss of PAECs via BMPR2 mutations contributes to a pro-inflammatory state that would allow for immune cell infiltration, PASMC hyperproliferation, and occlusion of the vessel lumen, which is observed in PAH histology [20]. Activation of nuclear factor erythroid 2-related factor 2 (Nrf2) has been shown to improve mitochondrial function and reduce the generation of ROS and inflammation, while also reducing arterial and RV remodeling [151,152]. Currently, bardoxolone methyl, an inducer of Nrf2, is underway in a phase II clinical study in PAH patients (NCT02036970) [152]. Taken together, the relationship among mitochondrial biogenesis, fusion, fission, and function are important in the observed hyperproliferation observed in PAH.

The upstream mechanisms underlying alterations in mitochondrial structure and metabolic reprogramming are unclear. One regulator, the deacetylase Sirtuin 3 (SIRT3), organizes multiple levels of mitochondrial function through mitochondrial protein deacetylation and thus activation of enzymes and ETC complexes [153]. A single nucleotide polymorphism (SNP) in the *SIRT3* gene led to a decrease in enzymatic activity and was associated with the development of metabolic syndrome in humans [154]. Thus, decreased expression or function of SIRT3 could serve as an explanation for the metabolic abnormalities in PAH. Mouse PASMCs deficient in SIRT3 revealed hyper-acetylation, which presumably caused the decreased PDH activity and depletion of TCA cycle intermediates. Interestingly, genetic ablation of SIRT3 also caused the activation of NFAT, the previously discussed transcription factor that promotes PH pathogenesis [155]. The traditional MCT-induced rat model of PAH demonstrated attenuation of *PGC1α and SORT3* mRNA levels, which when coupled with the finding that *SIRT3* KO mice developed spontaneous PAH, further implicates SIR3 as having a broad regulatory role in the events underpinning PAH metabolic reprogramming [155]. Of note, PGC-1α can activate *Sirt3* gene expression, which was previously discussed as being dysregulated downstream of BMPR2 mutations [148,156].

### 3.9. Targeting Endoplasmic Reticulum Stress

Dysregulation of calcium ion dynamics within the cell has the potential to manifest as endoplasmic reticulum (ER) stress. Intracellular calcium is one of the most important intracellular signaling molecules, requiring tight regulation in time, space, and concentration for proper homeostatic functioning of the cell [157]. The ER serves a critical homeostatic function in the storage of intracellular calcium and the maintenance of its homeostasis [158]. Furthermore, the mitochondrion, as an organelle with tightly controlled calcium dynamics, is dependent on the ER for maintenance of calcium homeostasis and proper functioning of calcium-dependent mitochondrial enzymes such as PDH, α-ketoglutarate dehydrogenase, and isocitrate dehydrogenase [15,159]. As previously discussed, the MPTP opens in response to depolarization and ROS to mediate the translocation of proapoptotic mediators [130]. An inability of calcium transfer between the ER and mitochondria would predispose mitochondria to remain in a relatively hyperpolarized state, conferring resistance to apoptosis and contributing to the observed phenotype seen in PAH [131].

The transfer of calcium from the ER to mitochondria is dependent on the tubular structure of the ER in forming a functional ER-mitochondrial unit, which is critically regulated by the protein Nogo [23]. Specifically, the isoform Nogo-B, the only isoform found in pulmonary vasculature, is implicated as having a role in the disruption of the ER-mitochondria unit [160,161]. The upregulation of Nogo-B in mice SMCs is seen under conditions of ER stress induced by hypoxia via activation of the unfolded protein response (UPR) through activating transcription factor 6 (ATF6)—one of three prongs of the UPR [160,162]. The dysregulation of calcium dynamics predisposes the cell to increased unfolded or misfolded proteins, resulting in ER stress and activation of the UPR—a fundamentally adaptive cytoprotective mechanism meant to overcome cellular stress via the upregulation of relevant chaperones and the global shutdown of protein synthesis [163]. Increased Nogo-B widened the distance between the ER and mitochondria and decreased mitochondria-dependent apoptosis; however, genetic ablation in Nogo-A/B^−/−^ mice prevented the observed hypoxia-induced changes, conferring resistance to PAH development [160]. Inhibition of ATF6 using the protease inhibitor 4-(2-aminoethyl)benzenesulfonyl fluoride hydrochloride (AEBSF) to prevent its cleavage and activation or using an ATF6 small interfering RNA prevented Nogo-B induction in PASMCs under ER stress and normalized Nogo-B expression in PAH PASMCs [160,164].

Chemical chaperones like 4-phenylbutyrate (PBA) and tauroursodeoxycholic acid (TUDCA) are other potential therapeutic agents capable of reducing ER stress. Administration of PBA prevented and reversed PAH in CH-induced mice and in MCT-induced rats. Moreover, PBA and TUDCA inhibited the ATF6 prong of the UPR in PASMCs under hypoxia, causing a subsequent decrease in Nogo expression, mitochondrial calcium levels, and mitochondrial membrane potential. Both in vitro and in vivo, these agents reduced proliferation and induced apoptosis in PASMCs, which reversed and prevented pulmonary vascular remodeling [165]. Interestingly, BMPR2 mutations have been implicated in protein misfolding, aggregation, and subsequent ER stress, and PBA treatment has been shown to restore BMPR2 signaling in HeLa cells transfected with the human disease-causing BMPR2 mutant [166]. Notably, PBA and TUDCA already have FDA approval for urea cycle and cholestatic liver disorders, meaning there is a potential for rapid clinical translation [167]. Another modulator of the UPR prong mediated through PKR-like ER kinase (PERK), salubrinal, prevented and attenuated MCT-induced PAH in rats [168].

The significance of the therapeutic potential underlying ER stress is that its stimuli are broad and diverse, meaning that therapeutic treatment may be broadly relevant for multiple subtypes of PH. For example, ER stress can be triggered via hypoxia, mutations in BMPR2, HIV or HSV infected PASMCs, and overexpression of Notch3—all of which have been linked to the pathogenesis of PAH [166,169,170,171,172]. Such capacity to integrate many disparate signals to mediate adaptive responses via mitochondria or other cytoprotective mechanisms implicates the ER as a convergence point in metabolic dysfunction.

### 3.10. Targeting Metabolic Dysfunction beyond the Pulmonary Vasculature: Fatty Acid Oxidation in the Right Ventricle

**Figure 2:**
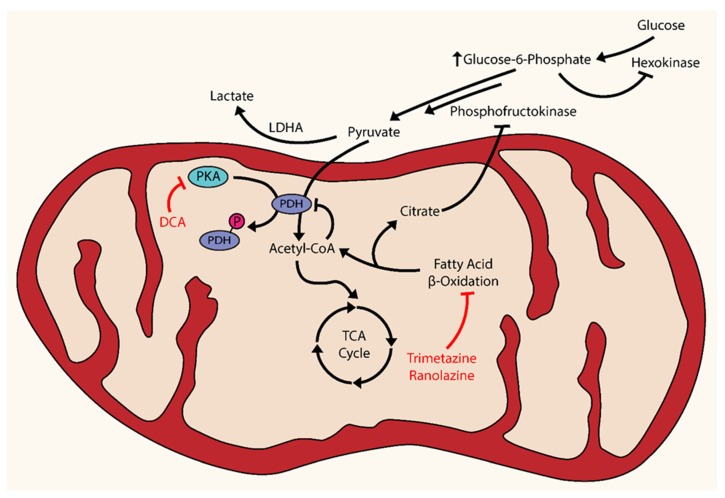
The Randle Cycle in Right Ventricular Dysfunction. The Randle cycle depicts a reciprocal, mutually-inhibitory relationship between glucose oxidation and fatty acid oxidation (FAO). The accumulation of acetyl-CoA and citrate from fatty acid β-oxidation inhibits pyruvate dehydrogenase (PDH) and phosphofructokinase, respectively. Moreover, the accumulation of glucose-6-phosphate inhibits hexokinase, further attenuating glycolysis. Inhibition of PDH, phosphofructokinase and hexokinase results in the inhibition of glucose oxidation; thus, FAO inhibits glucose oxidation in cardiomyocytes. Intervention with dichloroacetate (DCA) inhibits pyruvate dehydrogenase kinase (PDK)-mediated inhibition of PDH. The FAO inhibitors trimetazidine and ranolazine prevent acetyl-CoA and citrate accumulation, thereby preventing FAO-mediated shutdown of glucose oxidation.

The natural course of PAH is described as a pathological remodeling of pulmonary vessels that results in increased pulmonary vasculature resistance, causing increased afterload and strain, hypertrophy, dysfunction, and ultimately failure of the RV. Traditionally, the obvious therapeutic approaches for PAH sought to target the upstream events of this pathological cascade: pulmonary vascular remodeling and resistance. However, experimental evidence has demonstrated that therapeutic intervention targeting the RV may also be worthwhile, especially since RV function has shown to be a powerful prognostic factor over other measures of pulmonary vascular obstruction [173]. Positron emission tomography (PET) studies have shown an increase in glucose uptake by the RV in both animals and patients with RVH, which implies an upregulation of glycolysis and dysregulation of mitochondrial metabolism [174]. Under normal conditions, fatty acid oxidation (FAO) accounts for the vast majority (60%–90%) of energy production in cardiomyocytes, while the rest (10%–40%) is driven by GO [175]. Notably, there exists a mutually competitive relationship between FAO and GO, known as the Randle cycle, that can be exploited in the treatment of RV hypertrophy [176]. The Randle cycle acts through an increased production of citrate during FAO, which inhibits phosphofructokinase (PFK) and causing a subsequent accumulation of glucose-6-phosphate (G6P). The increase in G6P inhibits hexokinase, resulting in a decrease in the production of pyruvate. Pyruvate processing is further inhibited by the production of acetyl-CoA during FAO [177]. The cumulative effect of these metabolic events facilitates a preference toward FAO over GO in cardiomyocytes (Figure 2). In comparison to the LV, the RV has lower oxygen requirements in part due to lower mass and lower energy expenditure that is concordantly matched with lower coronary blood flow. Accordingly, RV hypertrophy and an increased metabolic demand coupled with less reservoir capacity of coronary blood flow implies a deficiency of oxygen despite a greater oxygen demand.

A loss of adequate oxygen supply causes activation of HIF-1α in cardiomyocytes and the upregulation of relevant glycolytic genes, which may account for the observed increased glucose uptake previously discussed [178]. In fact, the upregulation of HIF-1α in hypertrophied RV has been demonstrated in both CH-induced and MCT-induced models [179,180]. In both MCT- and pulmonary arterial banding (PAB)-induced RV hypertrophy, the administration of DCA reduced PDH phosphorylation, improved GO, partially restored Kv1.5 channel expression, and increased cardiac output and function [18]. These same beneficial effects were seen in the pulmonary vasculature, as previously mentioned [31,36]. The Randle cycle can also be exploited using FAO inhibitors to improve GO [181]. The FAO inhibitors trimetazidine and ranolazine can reverse the glycolytic shift back toward GO [182,183]. In a PAB-induced model, the use of trimetazidine and ranolazine reversed the metabolic changes in RV hypertrophy, enhanced GO, and improved RV function [184]. Accordingly, multiple clinical trials are currently underway or have been completed using FAO inhibitors.

A completed phase III study examined if three months of ranolazine treatment improved blood flow the heart, exercise capacity, and quality of life in symptomatic PAH patients with echocardiographic evidence of RV dysfunction (NCT01174173). At the end of the three months, there was an improvement in functional class, reduction and improvement of the RV, and an increasing trend toward better exercise capacity; however, there was no observed improvement in hemodynamic parameters [185]. A recently finished phase IV study examined the effects of six months of ranolazine in PH associated with LV diastolic dysfunction and has yet to be published (NCT02133352). Two ongoing studies sponsored by the University of Pennsylvania are examining the effects of ranolazine in subjects on stable PH therapies with RV dysfunction (RVEF < 45%) and comparing baseline metabolic profiles of subjects with and without RV dysfunction (NCT01839110 and NCT02829034). Additionally, a study is examining the differences in metabolism and functional imaging in PH patients with normal and persistent RV dysfunction. Metabolic and structural changes in response to treatment with ranolazine are also being measured with 11C-acetate and 18-FDG PET/CT imaging and cardiac MRI (NCT01917136). Lastly, a phase II trial has begun enrollment, studying the effects of trimetazidine on RV function, ventricular remodeling, and miRNA expression (NCT02102672). Overall, the relatively rapid time from the first proposal of FAO inhibitors as a treatment for PAH to their current use in multiple clinical trials shows the power behind the repurposing of currently approved therapies. Taken together, targeting dysfunction at the RV separately from dysregulation of pulmonary vascular remodeling provides another avenue, if used in combination to classical therapeutic approaches, in the treatment of PAH.

## 4. Future Directions

Since its original conception, the metabolic theory of PH has expanded in molecular scope and complexity outside the umbrella of the Warburg model. As a result, it serves as a powerful conceptual foundation for the elucidation of molecular dysregulation, disease manifestation, and future therapeutic intervention. As has been evidenced by the numerous multicenter, randomized, placebo-controlled clinical trials discussed in this review, strides are being made within the realm of metabolic intervention for PH; however, despite these successes, there are still barriers and challenges that must be overcome.

The current mainstay of PH treatment involves repurposed drugs that do more to support and preserve RV function than to halt or reverse disease pathology [14]. The lack of therapeutic agents capable of preventing and/or reversing structural alterations within the pulmonary vasculature and RV warrants the discovery of novel drug targets, such as the preclinical studies of novel drugs summarized in Table 1. Unfortunately, challenges in the identification of novel drug targets are inherent to the disease process of PH. As a relatively rare disease with multifactorial mechanisms of pathogenesis, identification of potential targets is variable based on the underlying disease-inciting stimulus. The elucidation of novel metabolic targets—when coupled with our recent understanding of genetic polymorphisms and their role in PH—becomes highly specific to subpopulations of patients, which further reduces an already limited population for clinical testing of new interventions.

Much of the rapid translation of preclinical findings in animal models to clinical trials in PH patients, as summarized in Table 2, can be attributed to the repurposing of already FDA-approved medications. Since the metabolic theory of PH share parallels in metabolic dysfunction with cancer, there is great potential for overlap and use of current therapies. In addition, the further development of new small molecules will be of critical importance, especially if targeting metabolic aberrations based on genetic polymorphisms, such as those downstream of BMPR2 mutations. The development of new small molecules will require detailed analyses of genomic and metabolomic screenings to identify polymorphisms and metabolites involved in disease manifestation and progression. The multitude of metabolic anomalies suggests that perhaps multiple errant pathways may need to be targeted for an efficacious clinical response. This rationale of combination therapy has been demonstrated in many chronic diseases, and long-term outcome trials are increasingly indicating that monotherapy is less effective than immediate combination therapy in delaying PH progression over time [186]. With many emerging metabolic therapies in clinical trials, there is an obvious need for studies that examine the combined effects of these new therapeutics.

Beyond therapeutics, the utility of metabolomic screening may also serve in the identification of novel clinical biomarkers for early diagnosis and prognosis. Mitochondria produce diffusible metabolites, which can serve as markers of disease progression, severity, and perhaps even signature when considering tailored therapies. As such, the use of precision medicine is central to the future of PH treatment, for the selective targeting of patient-specific pathways has the power of producing highly efficacious clinical responses and deepen our understanding of disease heterogeneity.

Noninvasive molecular imaging is an emerging application of technology in the study, staging, and potentially treatment of PH. The use of imaging can visualize metabolic shifts occurring in PH, such as enhanced glucose uptake and glycolysis following the inhibition of mitochondrial oxidative phosphorylation. The PET marker 18F-fluorodeoxyglucose (^18^FDG) is analogous to glucose and its uptake is driven by glucose transporters, meaning that its accumulation within cells can be visualized as a measure of cellular energy utilization. Diseased pulmonary vasculature in most patients with PAH has demonstrated via PET a chronic induction of the Warburg phenotype, as evidenced by increased glucose uptake; however, there is an observed variability among patients that is indicative of metabolic heterogeneity in patient populations [187,188]. Moreover, PET imaging of MCT-induced PAH rat model treated with DCA showed reductions in ^18^FDG uptake, revealing PET utility in investigating PH molecular pathology and its response to treatment [188]. The pulmonary vascular remodeling underlying PH involves ECs, SMCs, fibroblasts, and the infiltration of inflammatory cells, which limits the resolution of ^18^FDG PET imaging in being able to differentiate among cell types. Thus, increased ^18^FDG uptake is assumed to be due to a combination of a hyperproliferative state and an invasive inflammatory component [189,190,191]. Similar to other findings implicating the RV in the metabolic disease process, ^18^FDG uptake is markedly increased in the RV of both patients and rat models [18,174,192,193]. Future studies using PET can examine metabolism between two distinct anatomic compartments—the pulmonary vasculature and the RV—perhaps revealing previously undiscovered spatiotemporal relationships. Additional uses of PET can investigate drug distribution, drug target-binding, and drug-induced biochemical responses; however, its ease of use in preclinical and clinical application is unfortunately limited by cost and availability.

The complexity surrounding metabolite heterogeneity in PH patient populations can be addressed with novel applications of current technology, such as nuclear magnetic resonance, mass spectrometry, and chromatography. A recently published study utilized high-performance liquid and gas chromatography in combination with mass spectrometry in PAH and control populations to examine metabolic fingerprints. The study found a significant shift in metabolites related to the glycolytic shift and lipid-related energy imbalance that could be considered pathological hallmarks of PAH. In addition, novel metabolites not previously associated with PAH were discovered, suggestive of novel biochemical insight into metabolic dysfunction and potential use as clinical biomarkers [194].

## 5. Conclusions

The advancement of a metabolic theory in PH has been critical in the proposal of novel therapeutic strategies targeting metabolism and mitochondria. However, this theory has yet to comprehensively connect the seemingly discordant metabolic aberrations underlying PH pathophysiology. Understanding those fundamental molecular links will be essential in realizing the application of novel and potentially combinatorial therapies in the human population. For example, genomic and metabolomic profiles of PH patient populations will illuminate molecular explanations of individualized disease manifestations, allowing for the development of future treatment strategies based on a stratification of patient populations. Accordingly, due to the exponentially increasing possibilities of metabolic targets in PH, future work in the field will require not only further insight into underpinnings of the metabolic theory as a framework for molecular organization but also a better application of each person’s metabolic profile in tailoring and prioritizing specific therapeutic interventions.

## Figures and Tables

**Table 1:** Preclinical Interventions in Models of Pulmonary Hypertension. ATF6: activating transcription factor 6. BMPR2: bone morphogenetic protein 2. CH: chronic hypoxia. CO: cardiac output. DRP1: Dynamin-related protein 1. EC: endothelial cell. ER: endoplasmic reticulum. FAO: fatty acid oxidation. FHR: Fawn-Hooded rat. GLS1: glutaminase. HIF: hypoxia-inducible factor. HK1: hexokinase 1. KO: knockout. LDHA: lactose dehydrogenase A. MCT: monocrotaline. NFAT: nuclear factor of activated T cells. PAB: pulmonary arterial banding. PAP: pulmonary artery pressure. PASMC: pulmonary arterial smooth muscle cell. PDH: Pyruvate dehydrogenase. PVR: pulmonary vascular resistance. RV: right ventricle. RVH: right ventricular hypertrophy. RVSP: right ventricular systolic pressure. SOD: superoxide dismutase. VEGFR: vascular endothelial growth factor receptor.

Therapy	Finding	Citation
Dichloroacetate (DCA)	Restores PDH activity glucose oxidation; reverses MCT-induced PAH in rat model	[36]
	Reverses HIF activation, increases Kv1.5 channel expression; reduces PAH in FHRs	[31]
	Enhances activity and expression of Kv2.1 channels, improves hemodynamic parameters; prevents and reverses CH-induced PH in rat model	[21]
	Reduces PDH phosphorylation, increases glucose oxidation, restores Kv1.5 channel expression, and increases cardiac output and function in MCT- and PAB-induced RV hypertrophy	[18]
Ferric Carboxymaltose	Reverses consequences of iron deficiency, such as HIF upregulation, upregulation of *pdk1*, and decreases mitochondrial complex I activity	[57]
HDAC Inhibitors (Valproic Acid & Suberoylanilide Hydroxamic Acid)	Inhibit proliferative phenotype, exert anti-inflammatory effects, and reverse CH-induced PH in rats	[67]
Verteporfin	Decreases GLS1 expression and activity, decreases pulmonary arteriolar stiffness, reduces vascular remodeling, RVSP, and RV remodeling in MCT-induced PH	[80]
CB-839	Decreases GLS activity, proliferation, and pulmonary arteriolar remodeling in MCT-induced	[80]
5-aza-2’-deoxycytidine (5-AZA)	Restores SOD2 expression and mitochondrial function, inhibits PASMC proliferation, and increases cell apoptosis in vitro	[92]
Metalloporphyrin Mn(III)tetrakis (4-benzoic acid) porphyrin (MnTBAP)	Induces partial regression of PH, decreases vascular remodeling, and reverses the hyperproliferative phenotype in FHRs	[92]
Class I HDAC Inhibitors	Increases SOD3 expression and reduces proliferation of human idiopathic PAH PASMCs	[94]
Metformin	Inhibits PASMC proliferation in vitro; normalizes PAP and RVH in hypoxia- and MCT-induced PH in rats	[100]
	Reverses hypoxia-induced PH in mice	[102]
	Reduces PAP and reverses vascular remodeling in VEGFR blockade-induced PH rat model	[111]
VIVIT	Inhibits docking of calcineurin onto NFAT and thereby prevents its translocation	[134]
	Increases Kv1.5 channel expression, reduces intracellular potassium and calcium, mitochondrial membrane potential, and expression of bcl-2 in vitro	[117]
Cyclosporin A	Increases Kv1.5 channel expression, reduces intracellular potassium and calcium, mitochondrial membrane potential, and expression of bcl-2 in vitro; reduces PVR and PAP in MCT-induced rat model	[117]
Tacrolimus	Restores BMPR2 signaling in human PASMCs; reverses PH in a *Bmpr2* EC KO mouse model and in MCT-induced or VEGFR blockage/CH-induced rat models	[135]
Mdivi-1	Reduces DRP1 activation, mitochondrial fission, and PASMC proliferation; in vivo administration inhibits proliferation and improves exercise capacity and RV function	[142]
4-(2-aminoethyl)benzenesulfonyl fluouride hydrochloride (AEBSF)	Prevents nuclear translocation of ER stress-induced ATF6	[164]
4-phenylbutyrate (PBA)	Inhibits ATF6 in hypoxic PASMCs in vitro, decreasing expression of Nogo and restoration of mitochondrial calcium dynamics and function; reverses PH in CH-induced mice and MCT-induced rats	[165]
	Restores BMPR2 signaling in HeLa cells transfected with the human BMPR2 mutant	[166]
Tauroursodeoyxcholic Acid (TUDCA)	Inhibits ATF6 in hypoxic PASMCs in vitro, decreasing expression of Nogo and restoration of mitochondrial calcium dynamics and function	[165]
Salubrinal	Decreases lung macrophages, pro-inflammatory cytokines, PAP, and vascular remodeling in a MCT-induced PH rat model	[168]
Ranolazine	Stimulates glucose oxidation via PDH activation and reduces FAO in isolated rat hearts	[183]
	Increases CO and exercise capacity in PAB-induced PH rat model; increases oxygen consumption and ATP production; reduces expression of glycolytic mediators HK1 and LDHA	[184]
Trimetazidine	Enhances glucose oxidation via increased PDH activity and reduces FAO in rat hearts	[182]
	Increases CO and exercise capacity in PAB-induced PH rat model; increases oxygen consumption and ATP production; reduces expression of glycolytic mediators HK1 and LDHA	[184]

**Table 2:** Clinical Trials in Pulmonary Hypertension. 6MWD: 6-minute walk distance. DCA: dichloroacetate. ^18^FDG: 18F-fluorodeoxyglucose. PAP: pulmonary artery pressure. PAOP: pulmonary artery wedge pressure. PASP: pulmonary artery systolic pressure. PVR: pulmonary vascular resistance. MRI: magnetic resonance imaging. RVEF: right ventricular ejection fraction.

Therapy	Clinical Trial Identification	Design	Primary Endpoints	Treatment Duration	Status as of Publication
Dichloroacetate Sodium	NCT01083524	Phase I, interventional, open-label, non-randomized in idiopathic, familial, or anorexigen-associated PAH patients	Safety and tolerability of DCA	16 weeks	Completed September 2013 [unpublished]
Ferric carboxymaltose	NCT01288651	Phase IV, interventional, open-label, single group assignment in iron deficient patients with idiopathic PAH	Change in 6MWD	12 weeks	Completed [58]
Ferric carboxymaltose	NCT01847352	Single-blind, interventional, non-randomized in iron-deficient and iron-replete healthy volunteers	Change in PASP under subacute hypoxia with and without prior intravenous iron infusion	6 hours	Completed [59]
Ferric carboxymaltose (Europe) or Iron Dextran (China)	NCT01447628	Phase II, interventional, randomized, double-blind in patients with idiopathic, heritable, or anorexigen-associated PAH	Change in PVR and exercise capacity	12 weeks	Recruiting
Observing Low Fe-S Clusters	NCT02594917	Observational, cohort, prospective in patients with low Fe-S clusters	Change in 6MWD and PAP	—	Recruiting
Metformin	NCT01352026	Phase II, interventional, single group assignment, open-label in patients with PAH	—	—	Withdrawn due to lack of recruiting
Metformin	NCT01884051	Observational, cohort, prospective and Phase I, interventional in patients with idiopathic, heritable, scleroderma-, or anorexigen-associated PAH	Safety and tolerability of metformin (& secondary efficacy outcome measures)	—	Recruiting
Tacrolimus	NCT01647945	Phase II, interventional, randomized, double-blind in Group I PAH patients	Safety of low-dose tacrolimus (& 6MWD as secondary outcome)	18 weeks	Completed [unpublished]
Bardoxolone methyl	NCT02036970	Phase II, interventional, randomized, parallel assignment in PAH	Change in 6MWD	16 weeks	Ongoing
Ranolazine	NCT01174173	Phase III, interventional, single group assignment, open-label in patients with angina and PAH	Change in angina symptoms, 6MWD, and quality of life	3 months	Completed [185]
Ranolazine	NCT02133352	Phase IV, interventional, single group assignment, open-label in Group II PH patients	Change in mean PAP, PAOP, and PVR	6 months	Completed [unpublished]
Ranolazine	NCT01839110	Interventional, randomized, double-blind in subjects on stable PH therapies with RV dysfunction (RVEF <45%)	Number and percentage of subjects with high risk profile	26 weeks	Ongoing
Ranolazine	NCT02829034	Interventional, randomized, double-blind in subjects on stable PH therapies with RV dysfunction (RVEF <45%)	Percent change in RVEF as measured by MRI	26 weeks	Recruiting
Ranolazine ^11^C-Acetate ^18^FDG	NCT01917136	Phase II, interventional, single group assignment, open-label in PH patients with and without RV dysfunction	Change in myocardial oxygen consumption, ^18^FDG uptake, and myocardial perfusion	26 weeks	Ongoing
Trimetazidine	NCT02102672	Phase II, interventional, randomized, double-blind in Group I PAH patients	Changes in RV function assessed by echo 3d	3 months	Recruiting
